# Morphological Features of the Pygidial Glands and Chemical Composition of Their Secretions in Three Ground Beetle Taxa of the Tribe Chlaeniini (Coleoptera: Carabidae)

**DOI:** 10.3390/insects17070695

**Published:** 2026-07-03

**Authors:** Marija Vasović, Sofija Vranić, Marina Todosijević, Danica Pavlović, Nikola Vesović, Stefan Ivanović, Nina Ćurčić, Milan Radovanović, Ljubodrag Vujisić, Srećko Ćurčić

**Affiliations:** 1 Institute of Zoology, University of Belgrade – Faculty of Biology, Studentski Trg 16, 11000 Belgrade, Serbia; b3017_2018@stud.bio.bg.ac.rs (M.V.); sofija.vranic@bio.bg.ac.rs (S.V.); nikola.vesovic@bio.bg.ac.rs (N.V.); srecko@bio.bg.ac.rs (S.Ć.); 2 University of Belgrade – Faculty of Chemistry, Studentski Trg 12–16, 11000 Belgrade, Serbia; marinab@chem.bg.ac.rs; 3 Institute of Physics Belgrade, National Institute of the Republic of Serbia, University of Belgrade, Pregrevica 118, 11080 Belgrade, Serbia; danica.pavlovic@ipb.ac.rs; 4 University of Belgrade – Institute of Chemistry, Technology and Metallurgy, National Institute of the Republic of Serbia, Njegoševa 12, 11000 Belgrade, Serbia; stefan.ivanovic@ihtm.bg.ac.rs; 5 Geographical Institute “Jovan Cvijić”, Serbian Academy of Sciences and Arts, Đure Jakšića 9, 11000 Belgrade, Serbia; n.curcic@gi.sanu.ac.rs (N.Ć.); m.radovanovic@gi.sanu.ac.rs (M.R.)

**Keywords:** *Chlaenius*, pygidial glands, defensive secretions, BFM, NLM, GC–MS, NMR, 3-methylphenol

## Abstract

The relationship between the morphology of pygidial glands and the chemical nature of their secretions in the tribe Chlaeniini (family Carabidae) has long been recognised. We analysed the morphological features of the pygidial glands and the chemical composition of their secretions in three taxa: *Chlaenius* (*Chlaeniellus*) *tristis* (Schaller, 1783), *C*. (*Chlaenites*) *spoliatus spoliatus* (Rossi, 1792), and *C*. (*Chlaenius*) *festivus festivus* (Panzer, 1796). We examined the morphology of the pygidial glands in all three taxa using bright-field microscopy (BFM) and nonlinear microscopy (NLM). We used gas chromatography–mass spectrometry (GC–MS) and nuclear magnetic resonance (NMR) to analyse the chemical composition of the secretions. We measured and photographed the glands and conducted comparative morphological analyses. We detected a total of 21 chemicals in the pygidial gland secretions of the studied Chlaeniini. We found the highest number of compounds in *C*. *tristis* (17), slightly fewer in *C*. *festivus festivus* (13), and the lowest number in *C*. *spoliatus spoliatus* (seven). Thirteen compounds were new to the tribe Chlaeniini, eight of which were also new to the entire family Carabidae. The most dominant compound in the secretions of all three taxa was 3-methylphenol. We also discussed the taxonomic value of the chemical composition of the pygidial gland secretions.

## Simple Summary

Ground beetles possess a pair of defensive glands known as pygidial glands. The secretion from these glands acts as a repellent to various vertebrate and invertebrate predators. The repellent properties of the secretions result from the combined effect of different chemical compounds in the mixture. Pygidial glands and the compounds they produce are known to differ among ground beetle groups. For these reasons, we decided to morphologically examine the pygidial glands of three selected ground beetle taxa of the tribe Chlaeniini and to test chemically the defensive products of their secretions. Pygidial glands were studied using two types of microscopy, measured, and photographed. We found a relationship between the morphological features of the glands and the chemical nature of the secretions in the studied ground beetles. Among a total of 21 compounds, 3-methylphenol was the major chemical. Thirteen compounds were recorded for the first time in the tribe Chlaeniini, eight of which were new to the entire family Carabidae. Our results showed that, in some cases, along with other characters, the presence and combination of certain chemicals in the defensive secretion mixtures of ground beetles can be useful even in taxonomy.

## 1. Introduction

Insects can produce semiochemicals that elicit physiological or behavioural responses in individuals of the same or different species. The compounds involved in these behavioural responses can be categorised as pheromones, kairomones, or allomones [1]. Insect defence substances can be classified into more than 20 classes of organic compounds [2]. Ground beetles release their secretions under stressful conditions in one of three ways: oozing, spraying, or crepitation [3]. The released products consist of an active component with a strong, pungent scent designed to irritate and repel predators, and an adjuvant component that typically enhances the effectiveness of the active compounds [4].

The pygidial glands of ground beetles (Carabidae) produce chemicals that enable defence against predators [4,5]. These are paired organs located in the posterior part of the abdomen of ground beetles [6]. Morphologically, the glands consist of four parts: secretory lobes (i.e., secretory units), main collecting canal, reservoir, and efferent duct [6]. According to the classification of defence chemicals [3], ground beetles are capable of producing nine groups of organic compounds: hydrocarbons, aromatic aldehydes, aliphatic ketones, formic acid, saturated esters, phenols, benzoquinones, higher saturated fatty acids, and unsaturated carboxylic acids. However, recent studies show that many other classes of organic compounds, such as alcohols, nitriles, and monoterpenes, can also be synthesised by these glands [5].

Dierckx [7] provided the first visual representation of the pygidial glands in Chlaeniini ground beetles, using *Chlaenius* (*Chlaeniellus*) *nigricornis* (Fabricius, 1787), *C*. (*C*.) *nitidulus* (Schrank, 1781), *C*. (*C*.) *vestitus* (Paykull, 1790), and *C*. (*Chlaenius*) *festivus velutinus* (Duftschmid, 1812) as examples. The study described the secretory units of the first two species as spherical, while those in the third species were described as elongated. The last taxon was characterised by long, finger-like secretory units. More than half a century passed before research on the tribe resumed. Eisner et al. [8] presented the first photographic evidence of pygidial glands in the genus *Chlaenius* Bonelli, 1810, using *Chlaenius* (*Lithochlaenius*) *cordicollis* (Motschulsky, 1865) as the subject. The study noted that the morphological features of the glands were similar to those in *C*. *festivus velutinus* [7]. In subsequent years, research in this area continued, leading to the description of the pygidial glands in several other species. Kanehisa and Shiraga [9] presented the first scanning electron microscopy (SEM) micrographs of the pygidial glands of Chlaeniini, providing valuable insights into their external ultrastructure. To date, various authors have morphologically analysed the pygidial glands in 28 species of the tribe belonging to 12 subgenera, three genera, and two subtribes [6,9,10,11,12].

Eisner et al. [8] conducted the initial study on Chlaeniini defensive products, identifying 3-methylphenol in the pygidial gland secretion of *C. cordicollis*. The authors did not provide information on other secretion compounds but suggested that 3-methylphenol was not the only compound present. Moore and Wallbank [13] recorded an unsaturated hydrocarbon (not precisely identified) in the secretion of the Australian species *Chlaenius* (*Pelasmomimus*) *australis* Dejean, 1831, along with 3-methylphenol. Two subsequent studies analysed the secretions of 17 species of the genus *Chlaenius* and one species of the genus *Callistoides* Motschulsky, 1865, revealing a distinction between quinone- and phenol-secreting species of the tribe [9,10]. The same studies further suggested that the unidentified compounds in their samples could be aldehydes, ketones, esters, alcohols, or long-chain hydrocarbons. More recently, Holliday et al. [14] investigated geographical variation in the compounds present in the secretion of adult individuals of *C*. *cordicollis*. Holliday et al. [15] provided the first evidence of semiochemicals in the larval defensive glands of the same species. Finally, Holliday et al. [16] investigated age- and sex-related variation in the defensive secretion of adults of *C*. *cordicollis* and its role in sexual communication. Given the presence of several types of major compounds in the secretions of members of the tribe Chlaeniini [9,10], and the variation in other components of these secretion mixtures [14,16], it is important to focus not only on identifying the major compounds but also on other components in species that have not yet been chemotaxonomically analysed.

Particularly interesting is the study by Kanehisa and Shiraga [9], who investigated the relationship between the morphology of the pygidial glands and the nature of their products. The authors noted that the shape of the secretory units corresponds to the chemical class of the synthesised active compounds. The secretory lobes of ground beetles are either spherical, thick and elongated, or slender and finger-like in species producing carboxylic acids, quinones, or phenols, respectively. Based on the previous literature [6,7,8,9,10,11,12,13,14,15,16], members of the tribe Chlaeniini are capable of producing either quinones or phenols and exhibit the corresponding morphological features of the pygidial glands.

As no studies on pygidial gland morphology and chemical ecology of Chlaeniini have been conducted for over four decades and one decade, respectively, we decided to analyse the gland morphology and chemical composition of pygidial defensive gland secretions in three chlaeniine ground beetle taxa. For this purpose, we selected *Chlaenius* (*Chlaeniellus*) *tristis* (Schaller, 1783), *C*. (*Chlaenites*) *spoliatus spoliatus* (Rossi, 1792), and *C*. (*C*.) *festivus festivus* (Panzer, 1796). Another reason for choosing these taxa as models in this study is the limited knowledge of the morphology and anatomy of the glands in Chlaeniini, while compound detection methods are now much more precise than in previous decades [17]. Additionally, European species have received little attention in this context for a long time, which further motivated us to conduct this study. Kanehisa and Murase [10] investigated the morphology of the pygidial glands and chemically analysed their secretion in *C*. *spoliatus*, specifically in the subspecies *C*. (*Chlaenites*) *spoliatus motschulskyi* (Andrewes, 1928). Balestrazzi et al. [12] provided morphological and chemical data for the glands and their products in *C*. *festivus*, in the subspecies *C*. *festivus velutinus*. However, no one has morphologically examined the pygidial glands of *C*. *tristis*, *C*. *spoliatus spoliatus*, or *C*. *festivus festivus*. Regarding chemical analyses, Schildknecht et al. [18] previously studied *C*. *tristis*. The same study also investigated the secretion of its consubgener, *C*. *vestitus*, revealing the presence of phenols in the former species and quinones in the latter. These findings are consistent with the previously described morphology of the pygidial glands in *C*. *vestitus* [7], as the detected quinones correspond to the gland type reported in earlier studies. However, no comparable morphological data are available for *C. tristis*. Based on the chemical evidence, *C*. *tristis* is expected to possess finger-like lobes. However, all closely related taxa that were morphologically analysed [7] exhibited predominantly thick and elongated to spherical lobes.

Our intention was to determine how the morphological features of pygidial glands correlate with the chemical composition of their defensive secretions across the three selected ground beetle taxa of the tribe Chlaeniini, and whether these variations can serve as useful traits in taxonomy. Our working hypothesis was that there is a direct relationship between the morphological characteristics of the pygidial glands and the specific chemical profile of their secretions in the studied Chlaeniini taxa.

Our aims were: (i) to study the morphology of the pygidial glands in *C*. *tristis*, *C*. *spoliatus spoliatus*, and *C*. *festivus festivus* using bright-field microscopy (BFM) as the primary technique and nonlinear microscopy (NLM) as an additional method to provide more detailed insight into the gland ultrastructure [19]; (ii) to analyse the chemical composition of the glandular secretions of these taxa, with particular emphasis on the active compounds due to their potential taxonomic significance; and (iii) to compare our findings with those of earlier studies.

## 2. Materials and Methods

### 2.1. Study Objects

**Figure 1:**
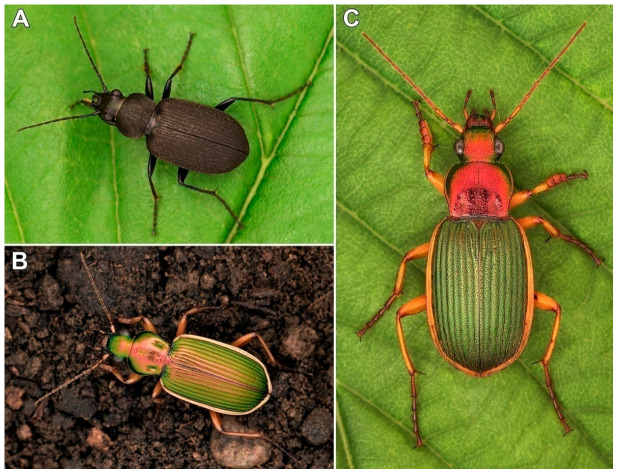
Habitus (dorsal view) of adult specimens of three analysed Chlaeniini ground beetle taxa: (**A**)—*Chlaenius tristis*; (**B**)—*Chlaenius spoliatus spoliatus*; (**C**)—*Chlaenius festivus festivus*. Photo: N. Vesović.

In this study, we selected three Chlaeniini taxa: *C. tristis*, a Palaearctic species distributed in Europe, northern Africa, and the Far East, inhabiting unshaded or partially shaded water edges with vegetation from lowlands to foothills (Figure 1A); *C*. *spoliatus spoliatus*, a Palaearctic subspecies inhabiting unshaded, overgrown margins of waters and swamps from lowlands to foothills (Figure 1B); and *C*. *festivus festivus*, a hygrophilous subspecies [20] distributed in central Europe, the eastern Mediterranean, west to southern France, the Caucasus, and central Asia [21,22] (Figure 1C). The mentioned subspecies are clearly distinct from related ones based on certain morphological characters (colouration of the abdominal sternites; punctuation of the head, pronotum, and elytral intervals; shape of the pronotum; and size of the transverse grooves on the pronotum) [21,22]. At the same time, they are the only subspecies of their respective species inhabiting Serbia.

### 2.2. Collecting and Handling Ground Beetles

We manually collected adult individuals of all examined taxa. We collected thirty-five individuals (10 males and 25 females) of *C*. *tristis* and 12 individuals (three males and nine females) of *C*. *festivus festivus* under stones and tree bark on the bank of the Tamiš River on 20 May 2023 near the city of Pančevo, Autonomous Province of Vojvodina, Serbia. We collected seven adult individuals (four males and three females) of *C*. *spoliatus spoliatus* at the same location on 10 August 2023. We kept the collected ground beetles in a portable plastic climate chamber under controlled laboratory conditions for several days until further analyses. These conditions included a constant temperature of 10 °C and high humidity. We used moist soil from the collection site and occasionally sprayed water into the chamber to maintain humidity. We fed the insects with earthworms.

### 2.3. Morphological Analyses of Pygidial Glands

We dissected the abdomens of three males and three females of each of the three taxa studied in 70% ethanol. First, we removed the connective tissue, and then carefully extracted the pygidial glands. We described and measured all morphological structures of the pygidial glands. After analysis, we deposited the dry ground beetle specimens in the collection of the Institute of Zoology, University of Belgrade—Faculty of Biology, Belgrade, Serbia (IZUB-FB).

#### 2.3.1. Bright-Field Microscopy (BFM)

We photographed the pygidial glands of the three taxa studied using a Nikon SMZ800N stereomicroscope (Nikon Corp., Tokyo, Japan) equipped with a Nikon DS-Fi2 camera (Nikon Corp., Tokyo, Japan), and measured various parts of the glands using a Nikon DS-L3 control unit (Nikon Corp., Tokyo, Japan). We conducted bright-field microscopy (BFM) at the IZUB-FB.

Figures were prepared using Zerene Stacker version 1.04 and Adobe Photoshop. Image processing included adjustments to brightness and contrast to enhance clarity, as well as cropping to optimise composition. Minor background imperfections and impurities were carefully removed to produce clean, visually consistent figures.

#### 2.3.2. Nonlinear Microscopy (NLM)

We performed nonlinear microscopy (NLM) imaging at the Institute of Physics Belgrade, National Institute of the Republic of Serbia, University of Belgrade, Belgrade, Serbia. We mounted the morphological structures of the pygidial glands on microscope slides with glycerin as the medium and covered each slide with a slip. We used a Mira 900-F femtosecond Ti-sapphire laser (Coherent Inc., Saxonburg, PA, USA), pumped by a Verdi V10 10-W laser (Coherent Inc., Saxonburg, PA, USA) at 532 nm, generating 160-fs pulses at a repetition rate of 76 MHz within a tuning range of 700–1000 nm to produce two-photon excitation fluorescence (TPEF). We used a Planachromat 20 × 0.8 NA microscope objective (Carl Zeiss, Jena, Germany). We detected only the natural autofluorescence of the gland samples, with the best signal obtained at 840 nm. We used VolView 3.4 open-source software (Kitware, Inc., Clifton Park, NY, USA) for three-dimensional (3D) visualisation of a series of slices using either volume rendering or maximum intensity projection algorithms.

### 2.4. Chemical Analyses of Pygidial Gland Secretions

#### 2.4.1. Chemical Extraction

We carried out sample preparation for gas chromatography–mass spectrometry (GC–MS) in the laboratory of the IZUB-FB at room temperature. We forced all collected specimens of each studied ground beetle taxon, whose age and physiological state were unknown, to spray their pygidial gland secretions into a 12-mL glass vial containing 0.5 mL dichloromethane (Merck, Darmstadt, Germany) by gently squeezing the abdominal tips. Milking was performed around noon. Each beetle was induced to release its secretion once. Analysis was independent of sex. The beetles’ natural ability to spray secretions at a distance was used to prevent contamination of the samples with cuticular components or substrate. To prevent oxidation and degradation of compounds, we subjected a portion of the extracts to GC–MS analysis immediately after preparation.

Sample preparation for nuclear magnetic resonance (NMR) spectroscopy was performed using a protocol analogous to that for GC–MS, with the only modification being the use of deuterated chloroform as the NMR solvent.

#### 2.4.2. GC–MS Analysis

We conducted analyses at the University of Belgrade—Faculty of Chemistry, Belgrade, Serbia, using a 7890A GC system (Agilent Technologies, Santa Clara, CA, USA) equipped with a 5975C inert XL EI/CI mass selective detector (MSD) (Agilent Technologies, Santa Clara, CA, USA) and a flame ionisation detector (FID) (Agilent Technologies, Santa Clara, CA, USA). We used a polar HP-INNOWax capillary column (30 m length, 0.25 mm inner diameter, 0.25 μm film thickness) (Agilent Technologies, Santa Clara, CA, USA).

We performed GC and GC–MS analyses in splitless mode for *C*. *tristis* and *C*. *spoliatus spoliatus*, and in 50:1 split mode for *C. festivus festivus*. For all analyses, the injection volume was 1 μL, the injector temperature was 220 °C, while the transfer line temperature was 280 °C. The carrier gas (He) flow rate was 2.0 mL min^−1^ at 40 °C. The column temperature was programmed to increase linearly from 40 °C to 240 °C at a rate of 10 °C/min, with a final 10-min hold for all analyses. The FID temperature was 300 °C. Electron ionisation (EI) mass spectra (70 eV) were acquired in the *m*/*z* range 35–550. The ion source and quadrupole temperatures were 230 °C and 150 °C, respectively.

We performed mass spectral deconvolution and extraction, retention indices (RIs) calculation and library search using the NIST AMDIS (Automated Mass Spectral Deconvolution and Identification System) software, version 2.70, and the commercially available NIST17 and Wiley07 libraries. RIs are obtained from the corresponding series of *n*-alkanes (analysed under the same chromatographic conditions). We calculated the relative percentages of the identified compounds from the corresponding gas chromatography–flame ionisation detection (GC–FID) peak areas using MSD ChemStation software, version E02.02 (Agilent Technologies, Santa Clara, CA, USA).

#### 2.4.3. NMR Analysis

We used NMR spectroscopy to analyse the secretion of *C*. *spoliatus spoliatus*. We recorded 1D and 2D NMR spectra [^1^H and^1^H-^1^H Correlation Spectroscopy (COSY)] on a Bruker 500 MHz spectrometer (Bruker BioSpin GmbH, Ettlingen, Germany) equipped with a 5-mm Broadband Inverse (BBI) probe head (Bruker BioSpin GmbH, Ettlingen, Germany). Deuterated chloroform containing 0.05% (*v*/*v*) tetramethylsilane (TMS) was used as the solvent. The spectra were referenced to the TMS signal.

## 3. Results

### 3.1. Morphological Characterisation of Pygidial Glands

**Figure 2:**
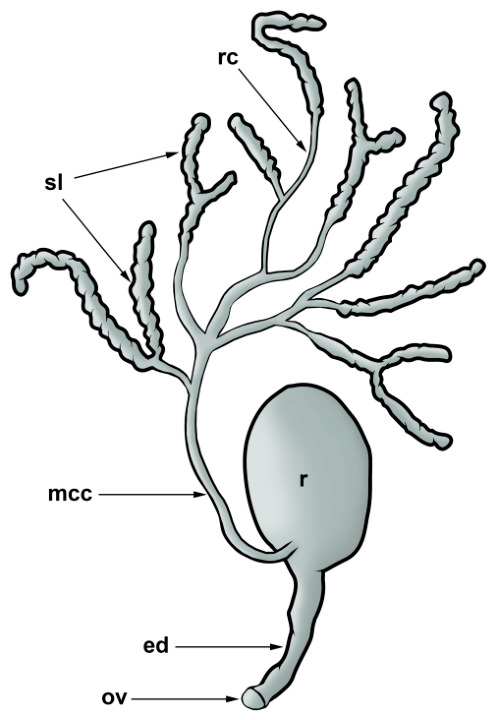
Appearance of the pygidial gland apparatus in Chlaeniini. Abbreviations: ed—efferent duct; mcc—main collecting canal; ov—opening valve; r—reservoir; rc—radial canal; sl—secretory lobes. Photo: N. Vesović.

**Figure 3:**
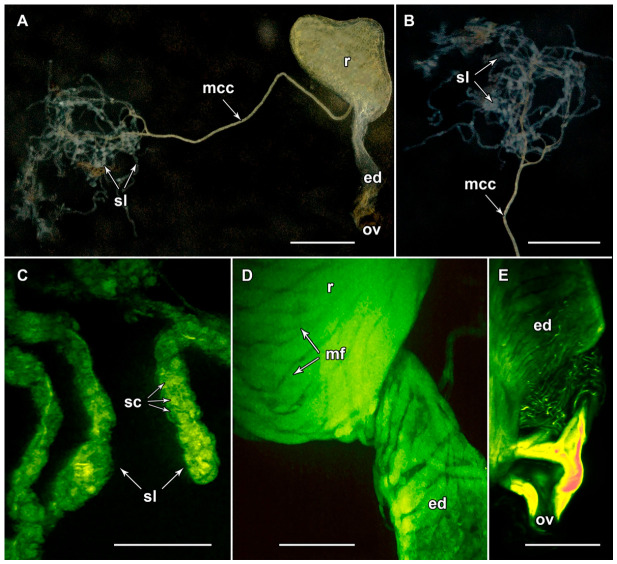
Morphology of the pygidial glands of *Chlaenius tristis* observed using BFM (**A**,**B**) and the TPEF modality of NLM (**C**–**E**): (**A**)—a single pygidial gland; (**B**)—secretory lobes and a main collecting canal; (**C**)—secretory lobes with secretory cells; (**D**)—part of a reservoir from which an efferent duct originates; (**E**)—an efferent duct and an opening valve. Abbreviations: ed—efferent duct; mcc—main collecting canal; mf—muscle fibres; ov—opening valve; r—reservoir; sc—secretory cells; sl—secretory lobes. Scales: 0.5 mm (**A**,**B**) and 100 μm (**C**–**E**).

**Figure 4:**
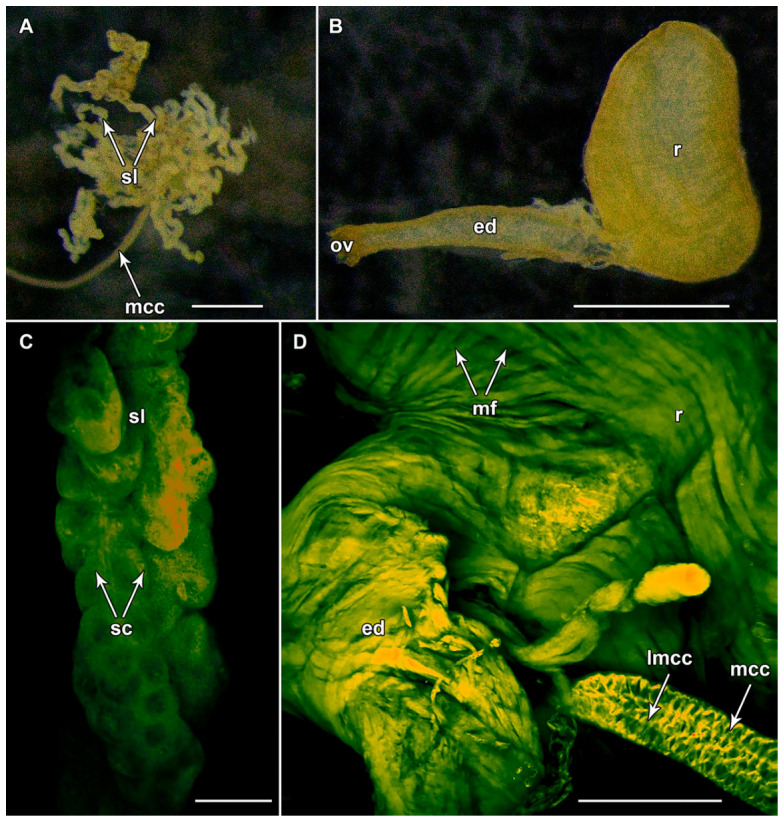
Morphology of the pygidial glands of *Chlaenius spoliatus spoliatus* observed using BFM (**A**,**B**) and the TPEF modality of NLM (**C**,**D**): (**A**)—secretory lobes and a main collecting canal; (**B**)—a reservoir and an efferent duct; (**C**)—a secretory lobe with secretory cells; (**D**)—part of a reservoir from which a main collecting canal and an efferent duct originate. Abbreviations: ed—efferent duct; lmcc—lumen of main collecting canal; mcc—main collecting canal; mf—muscle fibres; ov—opening valve; r—reservoir; sc—secretory cells; sl—secretory lobes. Scales: 1 mm (**B**), 0.5 mm (**A**), 100 μm (**D**) and 50 μm (**C**).

**Figure 5:**
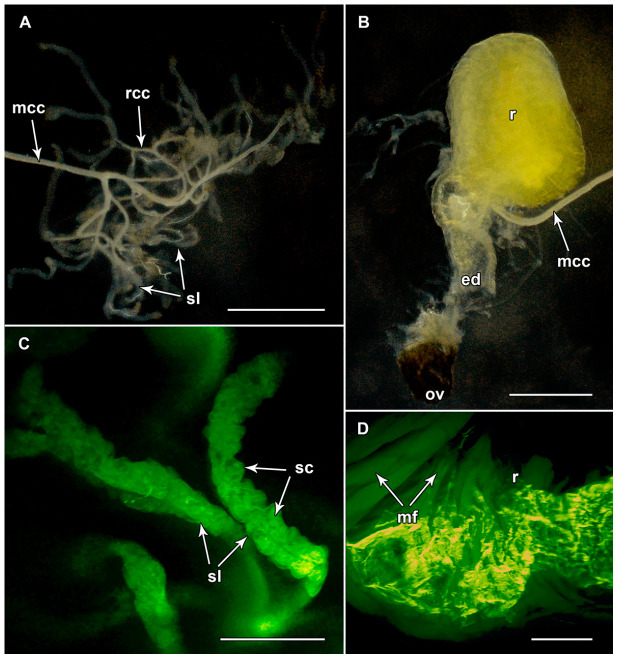
Morphology of the pygidial glands of *Chlaenius festivus festivus* observed using BFM (**A**,**B**) and the TPEF modality of NLM (**C**,**D**): (**A**)—secretory lobes, a radial collecting canal and a main collecting canal; (**B**)—a reservoir, a main collecting canal and an efferent duct; (**C**)—secretory lobes with secretory cells; (**D**)—part of a reservoir. Abbreviations: ed—efferent duct; mcc—main collecting canal; mf—muscle fibres; ov—opening valve; r—reservoir; rcc—radial collecting canal; sc—secretory cells; sl—secretory lobes. Scales: 0.5 mm (**A**,**B**) and 100 μm (**C**,**D**).

The morphology of the pygidial glands was analysed in three Chlaeniini ground beetles (Figure 2). In all examined *Chlaenius* taxa, the secretory lobes (i.e., secretory units) were elongated (Figure 3A–C, Figure 4A and Figure 5A,C) and consisted mainly of spherical secretory cells (Figure 3C, Figure 4C and Figure 5C). All secretory lobes of the glands formed a loose aggregation. The radial canals merged with the secretory lobes. One or more secretory lobes arose from a radial canal (Figure 3A, Figure 4B and Figure 5A,C). A single lobe was finger-like. Branching was observed in some lobes. In the latter case, it had a Y-shaped appearance (Figure 3B,C, Figure 4A,C and Figure 5A,C). Each lobe comprised several spherical secretory cells arranged around the radial canal. The main collecting canal branched into radial canals, which further divided into secondary or even tertiary branches (Figure 3A,B, Figure 4A and Figure 5A). The main collecting canal opened into the basal part of the reservoir, just above the initial part of the efferent duct (Figure 3A, Figure 4D and Figure 5B). The reservoirs of all three analysed taxa had a thick muscular layer (Figure 3D, Figure 4D and Figure 5D). There were no differences between the pygidial glands of males and females of the same taxon.

The length of the secretory lobes in *C*. *tristis* ranged from 0.60 to 1.78 mm. The width of the secretory lobes was about 20 μm (Figure 3A–C). Each aggregation contained 50 secretory units (Figure 3C). The radial canals measured 270–470 μm in length. The main collecting canal was 2.2 mm long, with an outer diameter of 20 μm and a lumen diameter of 10 μm. The reservoir was irregularly shaped, with a maximum length of 800 μm. Its maximum width was at the apex (740 μm), narrowing gradually towards the middle (710 μm) and base (200 μm) (Figure 3A). The efferent duct was approximately 870 μm long. Where the efferent duct exited the reservoir, its diameter was 140 μm, narrowing to about 70 μm in the median part. Near the opening valve, the efferent duct was approximately 140 μm wide. The opening valve was spherical (Figure 3E), with both length and diameter approximately 80 μm (Table 1).

The length of the secretory lobes of *C*. *spoliatus spoliatus* ranged from 420 to 830 μm, with an average of 600 μm. The width of the secretory lobes was 30 μm. There were 30–40 secretory units per aggregation (Figure 4A). The length of the radial canals (the first level of branching from the beginning of the main collecting canal) was 310 μm, with outer and inner diameters of 30 and 10 μm, respectively (Figure 4A). The length of the main collecting canal was about 2.7 mm, its outer diameter was about 60 μm, while the diameter of its lumen was about 20 μm (Figure 4D). The reservoir was ovoid (Figure 4B). Its maximum length was about 1.17 mm, with a maximum width of 800 μm in the middle. At the sides, it narrowed to 670 μm, while at the narrowest point it was 160 μm wide. The length of the efferent duct was approximately 1.33 mm. Where the efferent duct emerged from the reservoir, its total diameter averaged 270 μm, while at the median part it was about 210 μm. At the apical part, the outer diameter of the efferent duct was approximately 200 μm (Figure 4A). The opening valve was ovoid, with a length of about 210 μm and a diameter of about 180 μm (Table 1).

The length of the secretory lobes of *C*. *festivus festivus* ranged from 310 to 560 μm, with an average of about 450 μm. The width of the secretory lobes was approximately 30 μm. There were 25–35 secretory units per aggregation (Figure 5A,C). The length of the first-order branches of the radial canals was about 330 μm. The outer diameter of the radial canals was about 20 μm, while the diameter of their lumen was 8 μm. The main collecting canal was 2.80 mm long, with both the outer diameter and lumen diameter approximately 20 μm (Figure 5A,B). The reservoir was ovoid (Figure 5B), with a maximum length of approximately 700 μm and a maximum width of 550 μm at the median point. The efferent duct was 690 μm long, with width varying along its length. The diameter at the base was 260 μm, at the median part approximately 460 μm, and at the apex 90 μm. The opening valve was ellipsoidal (Figure 5B), with a length of about 80 μm and a diameter of 40 μm (Table 1).

### 3.2. Chemical Characterisation of Pygidial Gland Secretions

#### 3.2.1. GC–MS Analyses and Identification of Chemicals

**Figure 6:**
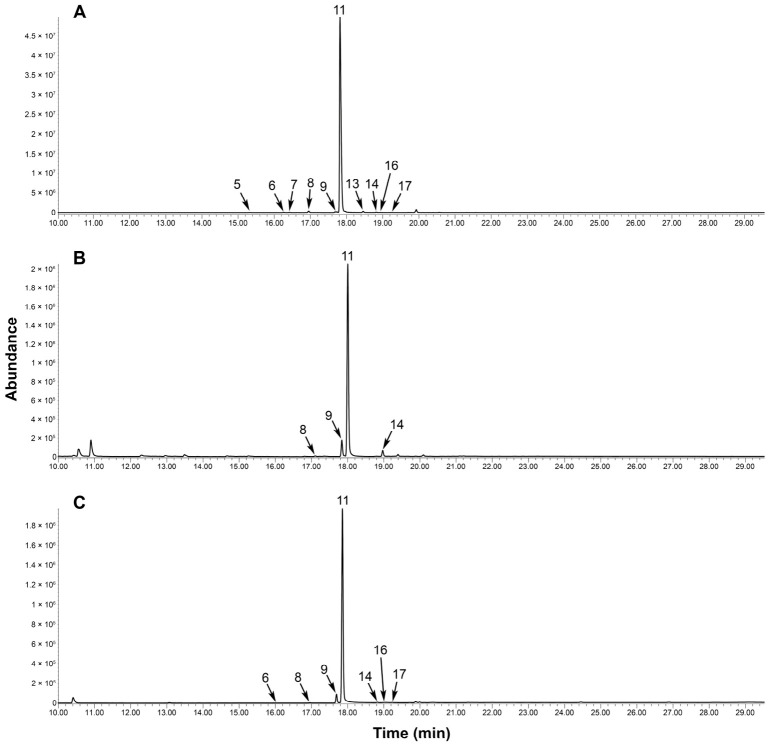
Chemical composition of the pygidial gland secretions of three ground beetle taxa analysed by GC–FID and GC–MS. (**A**)—*Chlaenius tristis*; (**B**)—*Chlaenius spoliatus spoliatus*; (**C**)—*Chlaenius festivus festivus*. The ordinal numbers of the peaks correspond to those in Table 1. Chromatogram annotations are restricted to phenolic compounds.

The GC–MS analysis of all three *Chlaenius* taxa (Figure 6A–C) reveals a total of 21 compounds in the secretions of the pygidial glands (Table 2).

In the secretion of *C*. *tristis*, we identified 17 compounds. The most abundant group of chemicals was phenols (10), followed by hydrocarbons (five), esters (one), and alcohols (one) (Table 2; Figure 6A).

**Figure 7:**
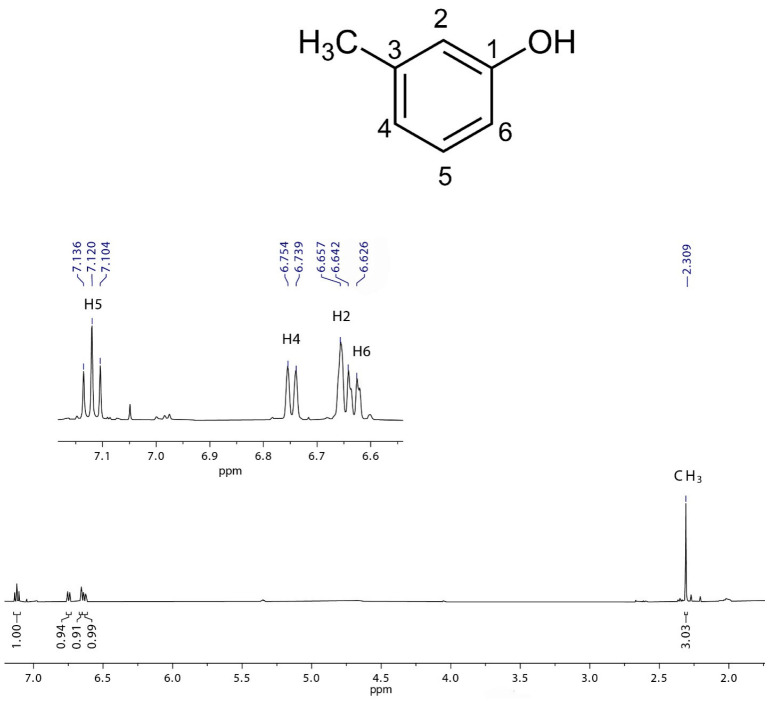
Expansions of the ^1^H NMR spectrum (500 MHz, CDCl_3_) of *Chlaenius spoliatus spoliatus*, with assignment of the signals of 3-methylphenol.

We isolated a total of seven compounds from the secretion of *C*. *spoliatus spoliatus*, with phenols (four) predominating, followed by hydrocarbons (three) (Table 2; Figure 6B and Figure 7).

We identified a total of 13 compounds in the secretion of *C*. *festivus festivus*. The most abundant group of chemicals was phenols (seven), followed by hydrocarbons (five), and alcohols (one) (Table 2; Figure 6C).

We found no qualitative or significant quantitative differences in the chemical composition of pygidial gland secretions between males and females of the same taxon.

#### 3.2.2. NMR Analyses and Identification of 3-Methylphenol

The ^1^H NMR spectrum of the CDCl_3_ extract of *C*. *spoliatus spoliatus* (Figure 7 and Figure S1) showed four aromatic signals (in the range δ 6.6–7.2) and a singlet of the methyl group at δ 2.31. Analysis of the coupling patterns among the aromatic signals confirms that the major component of the secretion is 3-methylphenol (*m*-cresol) [23]. The aromatic signal at δ 6.66 (br. s) was assigned to proton H2; the triplet at δ 7.12 (t, *J*_H5-H6_ 8 Hz, *J*_H5-H4_ 8 Hz) was assigned to H5; the signal of H4 was identified as a broad doublet at δ 6.75 (br. d, *J*_H4-H5_ 8 Hz); and the signal of proton H6 at δ 6.63 (br. d, *J*_H6-H5_ 8 Hz). In addition, 2D NMR COSY spectrum shows correlations between signals H5 and H4, H5 and H6 (Figure S2), as well as long-range coupling of the methyl group with both H4 and H2 signals.

## 4. Discussion

### 4.1. Morphological Aspects of Pygidial Glands

In addition to Chlaeniini, members of the tribe Panagaeini also secrete phenols and therefore share the corresponding morphological features of the secretory lobes (i.e., finger-like appearance) [10]. This indicates a close phylogenetic relationship between these two tribes, which was also confirmed by a recent study of the mitochondrial genome [24]. The only exception is *Tefflus* sp., whose secretory lobes are spherical, in contrast to those of other Panagaeini [6].

The pygidial glands of the taxa examined in this study are morphologically similar to some described in previous research on the tribe Chlaeniini [6,10,12]. According to the literature, Chlaeniini species have 30–50 secretory units per aggregation [7,9,11]. The number of secretory units varied among the taxa studied here. *Chlaenius tristis* had the highest number of secretory units per aggregation (50), followed by *C*. *spoliatus spoliatus* (30–40), while the lowest number was found in *C*. *festivus festivus* (25–30). The average length of the secretory units was significantly greater in *C*. *tristis* (1.02 mm) than in *C. spoliatus spoliatus* (600 μm) and *C. festivus festivus* (450 μm). The arrangement of the secretory cells around the radial collecting canal and finger-like shape of the secretory units in the representatives studied here are consistent with those observed in previous studies on phenol-secreting species of the tribe Chlaeniini [6,12]. The length of the units varied significantly even within a single gland. The greatest range in lobe length was recorded in *C*. *tristis*. The evolutionary factors or adaptive significance of this feature remain unclear. It may represent a universal characteristic of the group. However, as no precise measurements are available for other closely related species, it is currently not possible to draw any conclusions.

The previously analysed *Chlaenius* subspecies, *C*. *festivus velutinus*, had 50 secretory lobes per aggregation [12], while the closely related subspecies investigated here, *C*. *festivus festivus*, had only 25–35 units per aggregation. In the pygidial glands of the subspecies *C*. *spoliatus motschulskyi*, 20–49 secretory units per aggregation were present [10]. We did not observe such extreme variation in the number of secretory units in the closely related subspecies *C*. *spoliatus spoliatus* (45–50 lobes per aggregation), which corresponds to the upper limit reported for the former subspecies.

Several authors have noted differences in the shape of the secretory lobes among phenol-secreting, quinone-secreting, and carboxylic acid-secreting ground beetle species, which are slender and finger-like, thick and elongated, and more or less spherical, respectively [5,6,10,25]. The last group comprises the majority of ground beetle species. According to Forsyth [6], the secretory lobes of the pygidial glands of *C*. *vestitus* are thick and cylindrical, consistent with the quinone-based nature of its secretions [26]. In contrast, the pygidial glands of its phenol-producing consubgener, *C*. *tristis*, display morphological features consistent with those of phenol-producing species (i.e., finger-like secretory units) [10,26]. The chemical and morphological differences between these two species suggest that the monophyly of the group is questionable [26]. The deviation of *C*. *tristis* from the subgenus’s chemical and morphological profile raises doubts about its taxonomic status within the group. This interpretation is further supported by the recent findings of Kabak and Liang [27], who transferred the species to the subgenus *Achlaenius* Mandl, 1992, relying mainly on the structure of the apical gonocoxite.

The observed position of the entry point of the main collecting canal into the reservoir in the three analysed Chlaeniini representatives is consistent with that reported for previously studied members of the tribe [6,10,12]. The mean radial canal length was greatest in *C*. *tristis* (370 μm), and somewhat shorter in *C*. *festivus festivus* (330 μm) and *C*. *spoliatus spoliatus* (310 μm). The main collecting canal was longest in *C*. *festivus festivus* (2.8 mm) and *C*. *spoliatus spoliatus* (2.7 mm), and shortest in *C*. *tristis* (2.2 mm). The diameter of the lumen of the main collecting canal was the same in *C. festivus festivus* and *C*. *spoliatus spoliatus* (20 μm), and slightly smaller in *C. tristis* (10 μm). The outer diameter of the main collecting canal was the same in *C*. *festivus festivus* and *C*. *tristis* (20 μm), but larger in *C*. *spoliatus spoliatus* (60 μm).

The reservoirs of previously analysed Chlaeniini representatives had an ovoid shape [6,9,12]. In our study, only the reservoir of *C*. *tristis* differed, displaying an irregular shape due to a small notch in the apical part. The reservoir of *C*. *spoliatus spoliatus* was longer (1.17 mm) than those of *C*. *tristis* (800 μm) and *C*. *festivus festivus* (700 μm). The reservoir in *C*. *spoliatus spoliatus* was also somewhat wider (800 μm) than in *C*. *tristis* (740 μm) and *C*. *festivus festivus* (550 μm). The observed variation in reservoir shape in *C*. *tristis* is particularly interesting from an evolutionary perspective, as reservoir shape is known to be unique to certain groups in some cases [6,13].

*Chlaenius spoliatus spoliatus* had the longest efferent duct (1.33 mm), followed by *C*. *tristis* (870 μm) and *C*. *festivus festivus* (690 μm). The diameter of the efferent duct was greater in *C*. *spoliatus spoliatus* (270 μm) than in *C*. *festivus festivus* (260 μm) and *C*. *tristis* (140 μm). The opening valve was longer in *C. spoliatus spoliatus* (210 μm) than in both *C*. *festivus festivus* and *C*. *tristis* (each 80 μm). The width of the opening valve was also greater in *C*. *spoliatus spoliatus* (180 μm) than in *C*. *tristis* (80 μm) and *C*. *festivus festivus* (40 μm). Considering the relative body-to-pygidial gland ratio, it is noteworthy that *C*. *spoliatus spoliatus*, which is nearly the same size as *C*. *festivus festivus* [22], possesses significantly larger glands. In contrast, *C*. *tristis*, although smaller in overall body size [22] than *C*. *spoliatus spoliatus*, has glands of proportionally similar size.

It is important to note that, although we provided measurements of the pygidial glands and comparisons across taxa, our sample size was limited and therefore insufficient for statistical analyses. These comparisons offer only preliminary insight.

### 4.2. Chemical Aspects of Pygidial Gland Secretions

Members of the tribe Chlaeniini can synthesise either benzoquinones or phenols [5]. Regarding their defensive products, a total of 28 species from 13 subgenera, three genera, and two subtribes have been studied to date [3,8,9,10,12,13,14,15,16,18,26], 23 of which produced 3-methylphenol [5,8,9,10,12].

In this study, we identified 13 chemical compounds for the first time in the secretions of the tribe Chlaeniini: hexadecyl acetate, tricosane, (*Z*)-9-tricosene, *n*-heneicosane, 1-heneicosene, 2,6-dimethylphenol, 3-propylphenol, phenol, 4-methoxy-3-methylphenol, 2-methoxyphenol, hexadecanol, 1-pentadecene, and 1-docosene (Table 2 and Table 3). Eight of these compounds—2,6-dimethylphenol, 3-propylphenol, phenol, 4-methoxy-3-methylphenol, 2-methoxyphenol, hexadecanol, 1-pentadecene, and 1-docosene—were new to the entire family Carabidae.

According to Moore [3], the dominant compounds in ground beetles were once considered uniform within each genus. However, both phenols and benzoquinones have been detected in the defensive secretions of European species of the genus *Chlaenius* [5]. This phenomenon apparently occurs even within the same subgenus. Specifically, *C*. *tristis* is known to produce phenols [17], while all its consubgeners that have been chemically tested previously produce benzoquinones [10,12,18].

Schildknecht et al. [18] documented the disparity in the chemical composition of pygidial gland secretions between *C*. *tristis* and other *Chlaeniellus* species. Our study confirmed that the morphology of the glands corresponds to the nature of the produced compounds [10], but deviates from that observed in other members of the subgenus. The recent reclassification of *C*. *tristis* to another subgenus [27] indicates that such deviations should draw attention to the taxonomic position of the species. Many studies have debated whether the chemical composition of defensive secretions in ground beetles can serve as a reliable taxonomic character [3,13]. Although decades of investigations confirm that some patterns exist [5,18,27], these findings remain of limited use. Occasional reports indicate more specific patterns in the distribution of certain compounds. Well-known examples include the presence of salicylaldehyde exclusively in members of the subtribe Calosomatina (tribe Carabini), which clearly distinguishes them from the closely related subtribe Carabina [5,8], and the occurrence of benzaldehyde solely in the subfamily Cicindelinae [28,29,30]. Nevertheless, one must consider that erroneous placement of *C*. *tristis* in a phenol-secreting subgenus would render this character uninformative. Many recent studies provide no evidence that defensive compounds have taxonomic value [5].

Taking into account all available data [5], benzoquinone-producing species within the genus *Chlaenius* appear to be much rarer and are restricted to the subgenus *Chlaeniellus*. Previously, Kanehisa and Murase [10] provided results of chemical analyses for three *Achlaenius* species distributed in the Far East, and demonstrated that representatives of this subgenus are indeed phenol-producing. These findings strongly support the reclassification of *C*. *tristis* from the subgenus *Chlaeniellus* to the subgenus *Achlaenius* [27]. Considering the other genera of the tribe Chlaeniini, benzoquinones have only been found in *Callistus lunatus* (Fabricius, 1775) [5] (Table 3). However, the chemical ecology of many other genera in this diverse tribe remains uninvestigated.

The most significant difference between the chemical composition of the defensive secretions of *C*. *spoliatus motschulskyi* [10] and *C*. *spoliatus spoliatus* was the relative percentage of 3-methylphenol (98% and 76% in the former and latter subspecies, respectively). In addition to 3-methylphenol, we detected six other compounds in our sample of *C*. *spoliatus spoliatus*. The lower percentage of 3-methylphenol and the greater diversity of detected compounds in our sample could have various causes, ranging from intersubspecific differences to the effects of different habitats or food types. However, it is almost certain that reanalysing *C*. *spoliatus motschulskyi* using more sensitive equipment would reveal additional compounds, which would be beneficial for understanding the relationship between the chemical profiles of these two subspecies.

The pygidial gland secretions of *C*. *festivus festivus* and *C*. *festivus velutinus* shared some similarities [12]. In both subspecies, the dominant compound was 3-methylphenol. While *C*. *festivus velutinus* contained only two additional active compounds (2,5-dimethylphenol and 3,5-dimethylphenol), *C*. *festivus festivus* contained six active compounds, one of which was 2,5-dimethylphenol, also present in the former subspecies. Notably, extracts of *C*. *festivus festivus* contained 3,4-dimethylphenol, whereas *C*. *festivus velutinus* produced 3,5-dimethylphenol [12]. The reason for these differences is unclear, but a preliminary assumption is that they might reflect variation at the subspecific level or originate from dietary differences and the specific metabolic state of the individuals analysed. The two alkanes documented in *C*. *festivus velutinus* [12] were also present in *C*. *festivus festivus*, along with three additional hydrocarbons. The authors also emphasised that an unidentified hydrocarbon and two unidentified aromatic compounds were present in the secretion of *C*. *festivus velutinus* [12]. This strongly suggests that reanalyses are necessary to establish a definitive pattern of distribution of active compounds among the species of the diverse tribe Chlaeniini.

*N*-pentadecane and 8-heptadecene, which we detected in the secretions of *C*. *spoliatus spoliatus* and *C*. *festivus festivus*, were previously reported in two *Chlaenius* species from Australia. Specifically, the former compound was also present in *Chlaenius* (*Pelasmomimus*) *greyanus* White, 1841 [13], while the latter was recorded in *C*. *australis* [3].

Alcohols have not previously been reported in the pygidial gland secretions of the tribe Chlaeniini [5]. 1-Hexadecanol was found in the defensive secretions of *C*. *tristis* and *C*. *festivus festivus*. Earlier studies reported alcohols in the secretions of members of several other ground beetle tribes: Carabini, Cicindelini, Dryptini, Pterostichini, and Sphodrini [5,31]. Research by Holliday et al. [16] first identified an acetate ester (3-methylphenylacetate) in Chlaeniini. In our study, another acetate ester, hexadecyl acetate, was found in the secretion of *C*. *tristis*. Esters are usually associated with formic acid and hydrocarbons [32] or benzaldehyde and hydrocarbons [31], but they are also produced together with phenols in the defensive secretions of *C*. *cordicollis* and species of the genus *Craspedophorus* Hope, 1838 [3].

According to Kanehisa et al. [33], phenols have a strong repellent effect on predators. Hydrocarbons, alcohols, and esters are present as minor compounds in the defensive secretions of the pygidial glands of ground beetles [5]. Previous research suggests that hydrocarbons and esters may enhance the penetration of major compounds through the lipophilic cuticle because of their non-polar nature [3,4,10].

According to Bousquet [34], the defensive secretions of some members of the tribe Chlaeniini are most similar to those of the tribe Panagaeini. These two tribes are characterised by the presence of 3-methylphenol as the major compound, along with other phenolic compounds in their defensive secretions, distinguishing them from most other ground beetle groups. Surprisingly, the defensive secretion of the cave-dwelling subspecies *Pheggomisetes globiceps ninae* S. Ćurčić, Schönmann, Brajković, B. Ćurčić & Tomić, 2004 (tribe Trechini) contains trace amounts of 4-methylphenol [35].

Reanalysis of the chemical composition of the defensive secretion of *C*. *tristis* was necessary due to the significantly lower sensitivity of the equipment used in the previous experiment [17,18]. Analyses of the two subspecies, *C*. *spoliatus spoliatus* and *C*. *festivus festivus*, and their comparison with other conspecifics provided valuable insight into the chemical ecology of these closely related taxa.

The pygidial glands of Adephaga are of ectodermal origin and are typically exocrine, like the cuticular glands, which have been much less studied [36,37]. Given that no analytical replicates or confirmatory tests were conducted, it is possible that some detected compounds originated from cuticular hydrocarbons, fat body tissue, or other non-glandular sources. Vranić et al. [38] reported multiple long-chain hydrocarbons in the defensive secretions of two Bembidiini ground beetle species and suggested that these may be of cuticular origin. Although Chlaeniini release their secretions by spraying, unlike Bembidiini who ooze them out [6], some of the detected long-chain hydrocarbons may also be derived from the cuticle. However, no data are available on the cuticular profiles of *Chlaenius* species, making it difficult to draw reliable conclusions about whether contamination has occurred in this case. Future research should compare cuticular chemicals and those from pygidial glands, even though overlap appears scarce so far; for example, some docosene forms have been found in the cuticular exudates of certain ground beetles [39]. Holliday et al. [14] highlight the need to recognise patterns of variation in defensive secretions in Chlaeniini, rather than characterising a species’ secretion in a typological manner, thus indicating the direction in which further investigations of the chemical ecology of the tribe Chlaeniini should proceed.

## 5. Conclusions

The morphology of the defensive glands in the three analysed taxa is consistent with previously published data and supports the “chemical anatomy” hypothesis. Further morphological investigation of other representatives of the tribe Chlaeniini would be valuable for establishing evolutionary patterns in secretory lobes and reservoir shape across the genus *Chlaenius*. Morphometric analyses of the morpho-anatomical structures of the glands would also be beneficial in future to improve understanding of the evolution of the defensive system within the group. This study provides the first evidence of a chemotaxonomic character at the generic level and shows that congeners differ fundamentally in this regard. Although the taxonomic value of defensive compounds may be controversial, recognising unusual deviations in a taxon’s chemical profile can still indicate the need for further investigation of its status. Given the absence of analytical replication and confirmatory testing, the reported chemical profile of the defensive secretions of the three Chlaeniini ground beetles studied should be regarded as preliminary. Further studies are required to confirm the presence of the reported compounds and to verify that they originate from the pygidial gland secretions rather than other sources.

## Figures and Tables

**Table 1:** Comparative data on the dimensions (mean values in mm) of pygidial gland structures in three Chlaeniini ground beetle taxa studied.

Taxon	Gland Structure														
	SL			RC		MCC				R			ED		
	N	Length	Width	N	Length	N	Length	Outer Diameter	Inner Diameter	N	Length	Width	N	Length	Outer Diameter
*C. tristis*	10	1.02	0.02	4	0.37	4	2.2	0.02	0.01	4	0.80	0.74	4	0.87	0.14
*C. spoliatus spoliatus*	26	0.60	0.03	5	0.31	4	2.7	0.06	0.02	4	1.17	0.80	4	1.33	0.27
*C. festivus festivus*	23	0.45	0.03	5	0.33	4	2.8	0.02	0.02	4	0.70	0.55	4	0.69	0.26

ED—efferent duct; MCC—main collecting canal; N—number of measurements; R—reservoir; RC—radial canal; SL—secretory lobe.

**Table 2:** Chemical composition of the secretions of the pygidial glands of three Chlaeniini ground beetle taxa analysed by GC–FID and GC–MS. Retention indices are obtained from the corresponding series of *n*-alkanes at polar capillary column.

Peak	Compound	RI	Relative Percentage (%)		
			*C*. *tristis*	*C*. *spoliatus spoliatus*	*C*. *festivus* *festivus*
1	*n*-Pentadecane	1500	-	4.3	4.0
2	1-Pentadecene **	1528	-	8.1	-
3	*n*-Heptadecane	1700	-	-	0.3
4	8-Heptadecene	1729	-	1.0	<0.1
5	2-Methoxyphenol **	1882	0.1	-	-
6	2-Methoxy-4-methylphenol	1965	0.1	-	<0.1
7	4-Methoxy-3-methylphenol **	1980	0.1	-	-
8	Phenol **	2027	0.7	0.3	0.1
9	2,5-Dimethylphenol	2088	0.3	5.9	3.9
10	*n*-Heneicosane *	2100	0.3	-	-
11	3-Methylphenol	2105	95.1	74.9	90.5
12	1-Heneicosene *	2116	0.7	-	-
13	2,6-Dimethylphenol **	2172	0.5	-	-
14	3-Ethylphenol	2212	0.2	2.2	0.2
15	1-Docosene **	2224	0.1	-	-
16	3,4-Dimethylphenol	2232	0.1	-	0.1
17	3-Propylphenol **	2256	<0.1	-	0.1
18	*n*-Tricosane *	2300	0.1	-	0.1
19	Hexadecyl acetate *	2305	<0.1	-	-
20	(*Z*)-9-Tricosene *	2319	1.4	-	0.6
21	1-Hexadecanol **	2381	0.1	-	<0.1

RI—retention index; * first occurrence in the tribe Chlaeniini; ** first occurrence in the family Carabidae.

**Table 3:** Review of Chlaeniini taxa chemoecologically analysed to date and the chemicals in their pygidial gland secretions (modified after [5]).

Taxon/Compound	1,4-Benzoquinone	2,3-Dimethyl-1,4-benzoquinone	2,5-Dimethyl-1,4-benzoquinone	2-Ethyl-1,4-benzoquinone	2-Methyl-1,4-benzoquinone	2,3-Dimethylphenol	2,5-Dimethylphenol	3,4-Dimethylphenol	3,5-Dimethylphenol	3-Ethylphenol	3-Methylphenol	2-Metoxy-4-methylphenol	2-Metoxy-5-methylphenol	3-Methylphenylacetate
*Callistus lunatus*	*			*	*									
*Callistoides deliciolus*											*			
*Chlaenius* (*Achlaenius*) *micans*											*			
*Chlaenius* (*A.*) *ocreatus*											*			
*Chlaenius* (*A*.) *variicornis*											*			
*Chlaenius* (*Chlaeniellus*) *circumductus*	*			*	*									
*Chlaenius* (*C*.) *inops*	*			*	*									
*Chlaenius* (*C*.) *prostenus*	*			*	*									
*Chlaenius* (*C*.) *tristis*											*			
*Chlaenius* (*C*.) *vestitus*	*	*	*		*									
*Chlaenius* (*Chlaeniostenus*) *circumdatus*											*			
*Chlaenius* (*C*.) *darlingensis*											*			
*Chlaenius* (*Chlaenites*) *spoliatus motschulskyi*											*			
*Chlaenius* (*Chlaenius*) *festivus velutinus*							*		*		*			
*Chlaenius* (*C*.) *pallipes*											*			
*Chlaenius* (*Epomis*) *nigricans*											*			
*Chlaenius* (*Haplochlaenius*) *costiger*											*			
*Chlaenius* (*Lissauchlaenius*) *naeviger*											*			
*Chlaenius* (*L*.) *posticalis*											*			
*Chlaenius* (*L*.) *tetragonoderus*											*			
*Chlaenius* (*Lithochlaenius*) *cordicollis*						*	*	*		*	*	*	*	*
*Chlaenius* (*L*.) *noguchii*											*			
*Chlaenius* (*Ocybatus*) *bioculatus*											*			
*Chlaenius* (*Pachydinodes*) *abstersus*											*			
*Chlaenius* (*P*.) *virgulifer*											*			
*Chlaenius* (*Pelasmomimus*) *australis*											*			
*Chlaenius* (*P*.) *greyanus*											*			
*Chlaenius* (*Trichochlaenius*) *chrysocephalus*											*			

* presence of compound.

## Data Availability

The original contributions presented in this study are included in the article. Further inquiries should be directed to the corresponding author.

## Supplementary Materials

The following supporting information can be downloaded at: https://www.mdpi.com/article/10.3390/insects17070695/s1, Figure S1: The ^1^H NMR spectrum (500 MHz, CDCl_3_) of the pygidial gland secretion of *Chlaenius spoliatus spoliatus*, with assignment of the signals of 3-methylphenol; Figure S2: The ^1^H-^1^H COSY NMR spectrum (500 MHz, CDCl_3_) of the pygidial gland secretion of *Chlaenius spoliatus spoliatus*, showing 3-methylphenol correlations; Figure S3: GC–FID chromatogram of the dichloromethane extract of pygidial gland secretion of *Chlaenius tristis*; Figure S4: GC–FID chromatogram of the dichloromethane extract of pygidial gland secretion of *Chlaenius spoliatus spoliatus*; Figure S5: GC–FID chromatogram of the dichloromethane extract of pygidial gland secretion of *Chlaenius festivus festivus*.

## Abbreviations

The following abbreviations are used in this manuscript:

AMDIS	Automated mass spectral deconvolution and identification system
BBI	Broadband inverse
BFM	Bright-field microscopy
COSY	Correlation spectroscopy
EI	Electron ionisation
FID	Flame ionisation detector
GC–FID	Gas chromatography–flame ionisation detection
GC–MS	Gas chromatography–mass spectrometry
IZUB-FB	Institute of Zoology, University of Belgrade—Faculty of Biology
MSD	Mass selective detector
NIST	National Institute of Standards and Technology
NLM	Nonlinear microscopy
NMR	Nuclear magnetic resonance
RI	Retention index
SEM	Scanning electron microscopy
TMS	Tetramethylsilane
TPEF	Two-photon excitation fluorescence
