# Supplementary material for: Morphological Features of the Pygidial Glands and Chemical Composition of Their Secretions in Three Ground Beetle Taxa of the Tribe Chlaeniini (Coleoptera: Carabidae)
Source: Insects. 2026 Jul 3;17(7):695. doi: 10.3390/insects17070695 (PMC13410220; doi:10.3390/insects17070695)
Supplement: Supplementary file 1 [file insects-17-00695-s001.zip › insects-4314787-supplementary.pdf]

Supplementary Material

Morphological features of the pygidial glands and chemical composition of their secretions in three ground beetle species of the tribe Chlaeniini (Coleoptera: Carabidae)

Marija Vasović [1], Sofija Vranić [1], Marina Todosijević [2], Danica Pavlović [3], Nikola Vesović [1], Stefan Ivanović [4], Nina Ćurčić [5], Milan Radovanović [5], Ljubodrag Vujisić [2]* and Srećko Ćurčić [1]

[1] Institute of Zoology, University of Belgrade - Faculty of Biology, Studentski Trg 16, 11000 Belgrade, Serbia
[2] University of Belgrade - Faculty of Chemistry, Studentski Trg 12–16, 11000 Belgrade, Serbia
[3] Institute of Physics Belgrade, National Institute of the Republic of Serbia, University of Belgrade, Pregrevica 118, 11080 Belgrade, Serbia
[4] University of Belgrade - Institute of Chemistry, Technology and Metallurgy, National Institute of the Republic of Serbia, Njegoševa 12, 11000 Belgrade, Serbia
[5] Geographical Institute "Jovan Cvijić", Serbian Academy of Sciences and Arts, Đure Jakšića 9, 11000 Belgrade, Serbia
* Correspondence: ljubaw@chem.bg.ac.rs

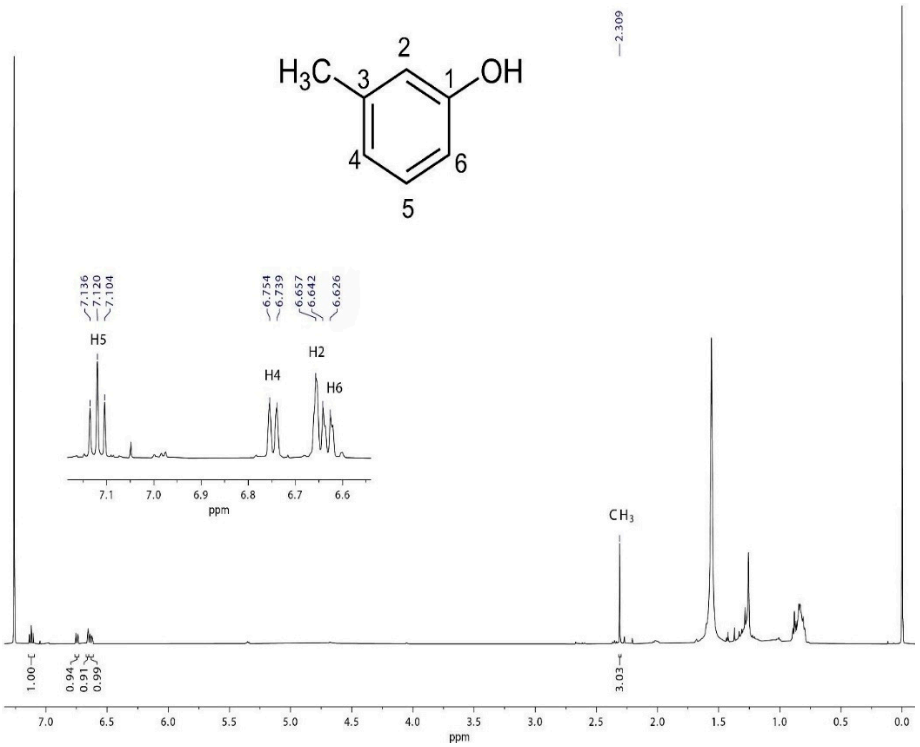

Figure S1. The ¹H NMR spectrum (500 MHz, CDCl3) of the pygidial gland secretion of *Chlaenius spoliatus spoliatus*, with assignment of the signals of 3-methylphenol.

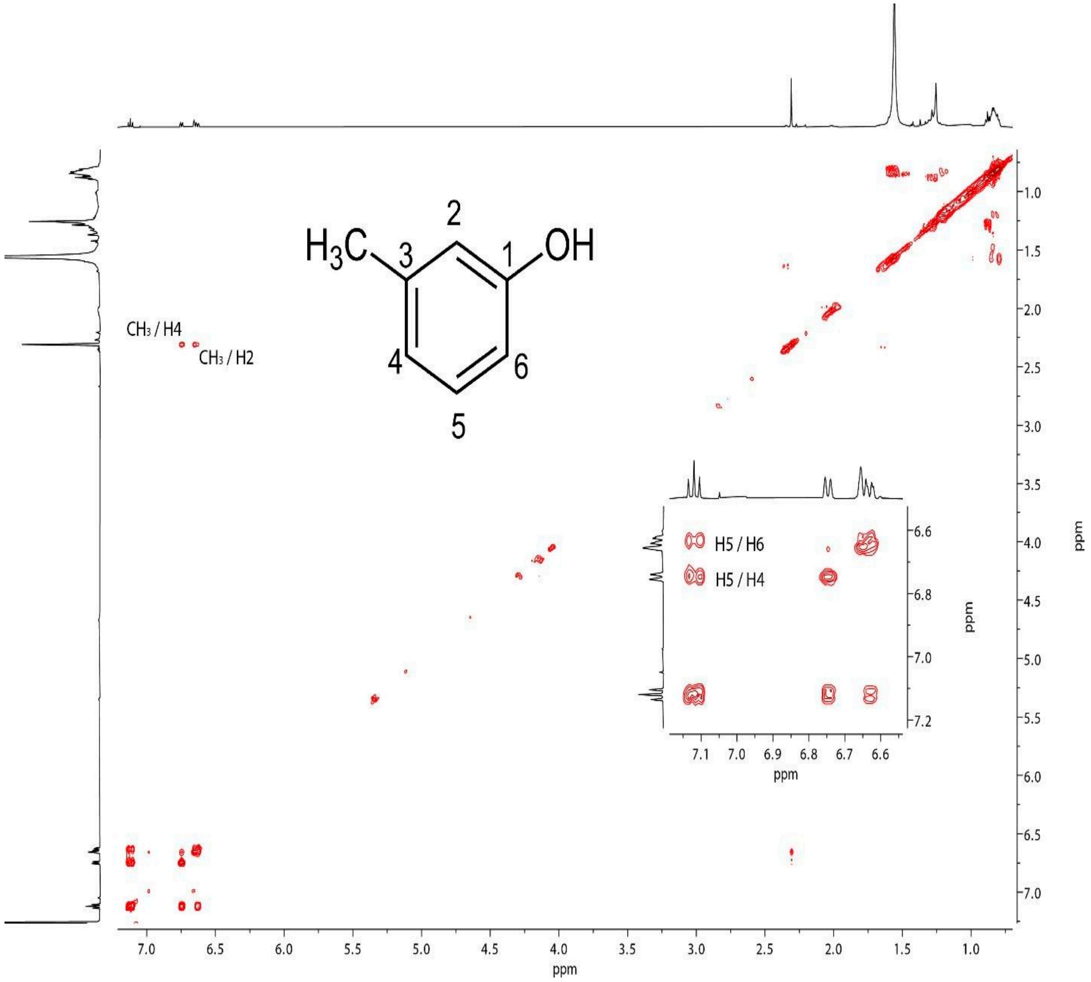

Figure S2. The ^{1}H-^{1}H COSY NMR spectrum (500 MHz, CDCl3) of the pygidial gland secretion of *Chlaenius spoliatus spoliatus*, showing 3-methylphenol correlations

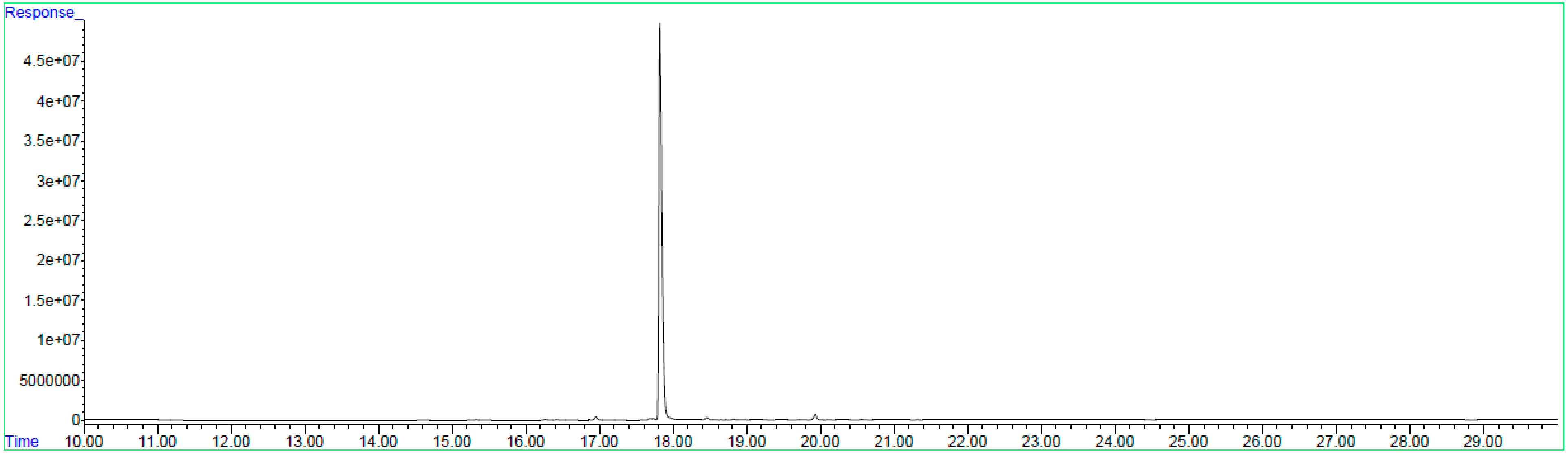

Figure S3. GC–FID chromatogram of the dichloromethane extract of pygidial gland secretion of *Chlaenius tristis*.

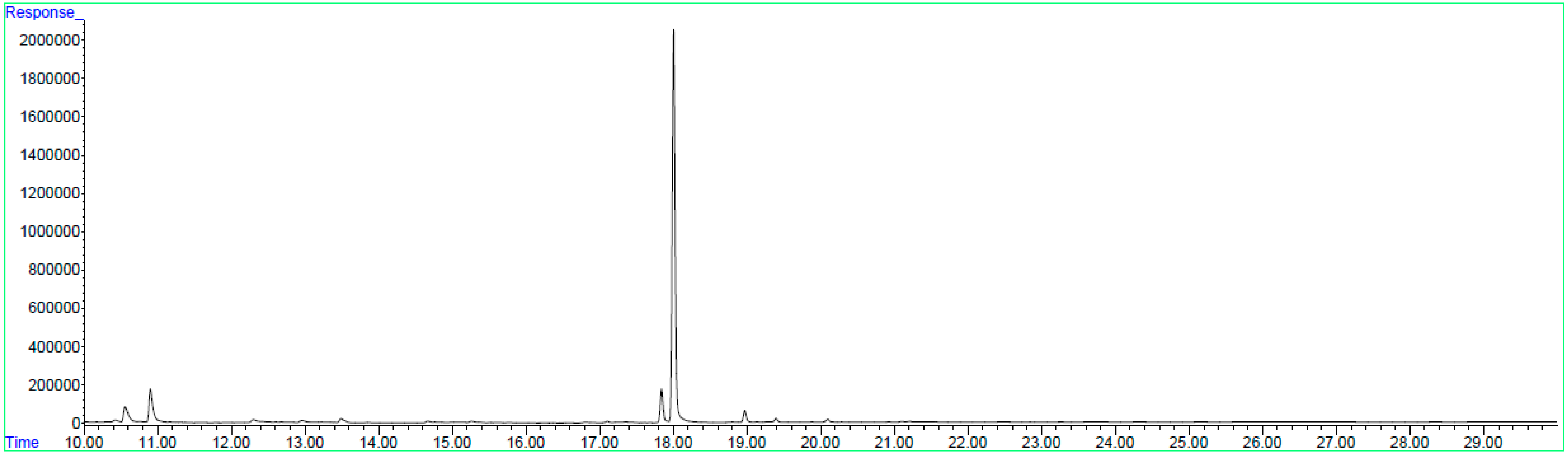

Figure S4. GC–FID chromatogram of the dichloromethane extract of pygidial gland secretion of *Chlaenius spoliatus spoliatus*.

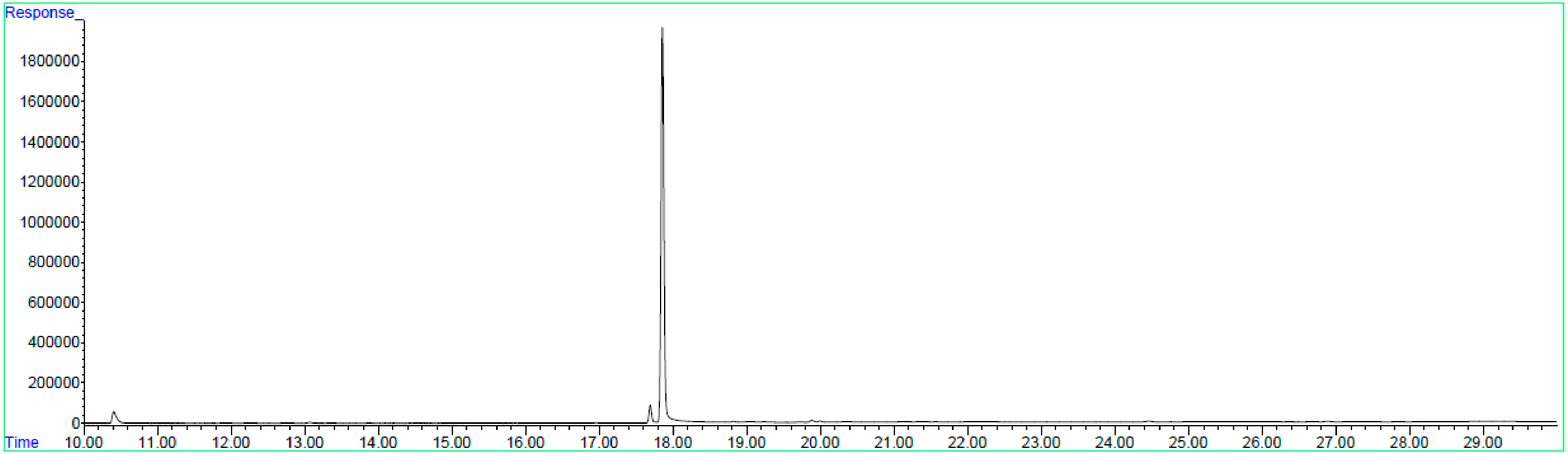

Figure S5. GC–FID chromatogram of the dichloromethane extract of pygidial gland secretion of *Chlaenius festivus festivus*.
